# MRI Findings Reflecting Ongoing Cardiac Arrest While Being on an MRI Scanner

**DOI:** 10.7759/cureus.54608

**Published:** 2024-02-21

**Authors:** Abdallah Q Al Khateeb, Pokhraj P Suthar, Sudeep Bhabad

**Affiliations:** 1 Department of Diagnostic Radiology and Nuclear Medicine, Rush University Medical Center, Chicago, USA

**Keywords:** syncope, mri, adult, pulseless electric activity, sudden cardiac arrest

## Abstract

Sudden cardiac arrest (SCA) is the abrupt loss of cardiac function that results in acute cardiovascular collapse and subsequent decreased or loss of various organ perfusion. Here, we present an interesting case of a 58-year-old man who developed abnormal brain MRI findings reflecting ongoing cardiac arrest due to pulseless electric activity (PEA) during an MRI scan. To our knowledge, this is the first case describing the MRI findings of ongoing cardiac arrest due to PEA. Our case is unique in imaging findings, which are not routinely encountered in day-to-day practice. This case raised awareness among the readers.

## Introduction

Sudden cardiac arrest (SCA) is the abrupt loss of cardiac function that results in acute cardiovascular collapse and subsequent poor cerebral and organ perfusion. Pulseless electric activity (PEA) describes the presence of spontaneous cardiac electric activity on electrocardiography (ECG) and the concurrent absence of blood flow sufficient to maintain consciousness and organ perfusion [1]. It was estimated that 17-28% of SCA patients present with PEA [2]. Here, we present an interesting case of a 58-year-old man who developed abnormal brain MRI findings reflecting ongoing cardiac arrest due to PEA while in the MRI scanner.

## Case presentation

A 58-year-old male presented to the emergency department with a single episode of syncope followed by a fall at home one hour ago in a room. On presentation, his vital signs were within normal range, except for tachycardia (heart rate of 115 beats per minute). The patient denied hitting his head while falling, having previous chest pain, or having shortness of breath. The Glasgow Coma Scale was 15/15. Apart from tachycardia and slurred speech, the rest of the physical and neurological examinations were within normal ranges. His previous medical history included hypertension, type 2 diabetes, schizophrenia, obesity, and a nicotine addiction disorder. There was sinus tachycardia with premature supraventricular complexes and fusion on electrocardiography. Noncontrast CT head demonstrated chronic microvascular ischemic changes without intracranial hemorrhage (images not shown). Laboratory workup in the emergency department revealed an elevated CPK, glucose, creatinine, leukocytosis with lactic acidosis, and hyponatremia (Table 1). Toxicology screening was unremarkable. The patient was started on supportive treatment with the correction of hyponatremia. A blood culture was obtained, and an intravenous empirical antibiotic was administered for sepsis. The patient was transferred to telemetry for continuous monitoring. After stabilization, magnetic resonance imaging (MRI) of the brain was planned to rule out an acute ischemic infarct.

**Table 1:** Laboratory values. eGFR: estimated glomerular filtration rate, NAAT: nucleic acid amplification test

	Laboratory value	Reference range
White blood count	14.38 K/uL	4.00 - 10.00 K/uL
Red blood count	3.76 M/uL	4.40-6.40 M/uL
Hemoglobin	11.9 g/dL	12.0-18.0 g/dL
Platelet count	278 K/uL	150-450 K/uL
Sodium	122 mmol/L	137-147 mmol/L
Potassium	4.1 mmol/L	3.4-5.3 mmol/L
Chloride	91 mmol/ L	99-108 mmol/ L
CO_2_ total	14 mmol/L	22-29 mmol/L
Anion gap	17	8-16
Blood urea nitrogen (BUN)	29 mg/dL	8-21 mg/dL
Creatinine	2.39 mg/dL	0.75-1.20 mg/dL
BUN/creatinine ratio	12.1	8.0-25.0 ratio
eGFR	29 mL/min/1.73 sq m.	>=90 mL/min/1.73 sq. m.
Glucose	169 mg/dL	60-99 mg/dL
Calcium	8.8 mg/dL	8.7-10.7 mg/dL
Troponin I	0.01 ng/mL	0.00-0.09 ng/mL
CPK (creatine phosphokinase)	878 U/L	10-205 U/L
Ethanol	Not detected	Not detected
Magnesium	1.1 Low	1.6-2.7 mg/dL
Phosphorus	3.5	2.5-4.6 mg/dL
COVID-19 (NAAT)	Negative	Negative

**Figure 1:**
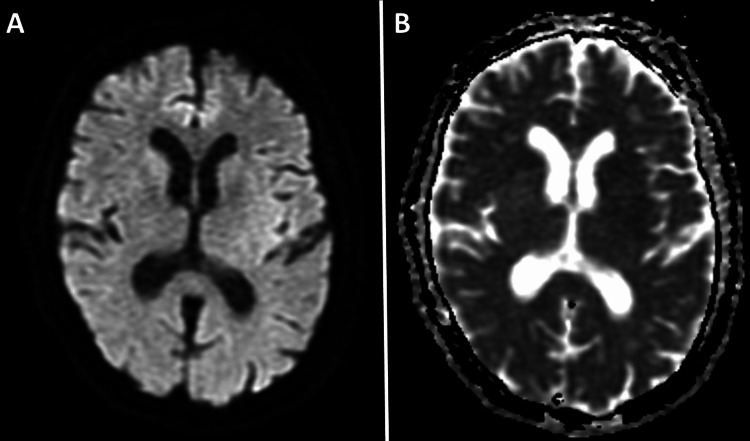
(A) Axial diffusion-weighted MRI of the brain (repetition time msec/echo time msec, 8500/100; flip angle, 90°; b value = 1000 sec/mm2) and (B) corresponding axial apparent diffusion coefficient maps (8500/100; flip angle, 90°; b value = 1000 sec/mm2) demonstrate the absence of restricted diffusion.

**Figure 2:**
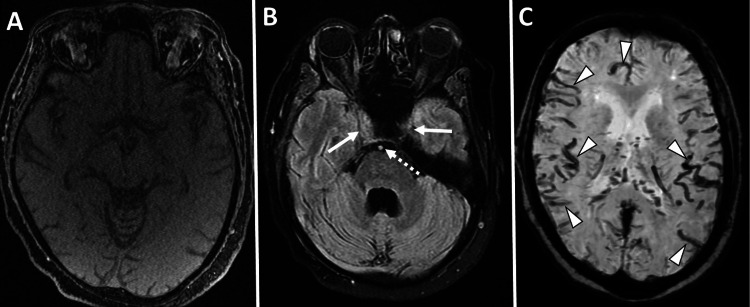
(A) Axial image of 3D time-of-flight magnetic resonance angiogram at the level of Sylvian fissures demonstrates a complete absence of flow-related signal within the intracranial circulation. (B) Axial fluid-attenuated inversion recovery MRI of the brain (inversion time 2500, repetition time msec/echo time msec, 9000/107, 5-mm section thickness) demonstrates loss of vascular signal void in the intracranial bilateral carotid arteries (solid white arrow) and basilar artery (dashed white arrow). (C) 3D susceptibility-weighted angiography (SWAN) imaging demonstrates serpiginous low signal intensities likely due to an intravascular buildup of deoxyhemoglobin and stagnant blood flow within most of the intracranial arterial and venous circulation (solid white arrowheads).

MRI of the brain was performed within four hours of admission. Earlier MRI sequences, such as diffusion-weighted and apparent diffusion coefficient maps, were reassuring in that they showed no evidence of restricted diffusion (Figure 1). However, a 3D time-of-flight MR angiogram disclosed the absence of flow within the anterior and posterior intracranial circulations (Figure 2A). At the same time, fluid-attenuated inversion recovery (FLAIR) imaging showed loss of the normal vascular signal void in the carotid and vertebrobasilar circulations and major venous structures, reflecting a slow blood flow (Figure 2B). In keeping with the global slow/stagnant arterial and venous blood flow, susceptibility-weighted angiography (SWAN) imaging revealed increased vessel visibility throughout the brain, correlating to stagnant flow and increasing intravascular deoxygenated blood (Figure 2C). During the real-time viewing of the images, clinical suspicion of circulatory arrest arose based on monitoring through a blood pressure cuff, EKG, and pulse oximetry, leading to the immediate initiation of a code for the patient. Pulseless electrical activity (PEA) was confirmed during CPR. Despite resuscitative efforts, the patient did not survive.

## Discussion

SCA is the abrupt loss of cardiac function that results in acute cardiovascular collapse and subsequent poor cerebral and multiorgan perfusion. Poor cerebral perfusion usually manifests as a complete loss of consciousness. PEA describes the presence of spontaneous cardiac electric activity on electrocardiography (ECG) and the absence of blood flow sufficient to maintain consciousness and organ perfusion [1]. It was estimated that 17-28% of SCA patients present with PEA [2]. ECG patterns commonly found in patients with PEA include wide, regular, or irregular QRS complexes [1]. This is the first reported case of a patient who suffered a PEA cardiac arrest while in the MRI scanner and the findings of his MRI brain were recorded.

After excluding other possible etiologies for the patient’s abnormal imaging findings, we concluded that SCA and acute circulatory collapse best explained them [2]. When the imaging started, the normal diffusion-weighted imaging (DWI) ruled out subclinical acute or subacute ischemia or infarction. On the subsequent TOF MRA acquisition, the global lack of intravascular flow-related signal was best explained by the slow or absent cerebral arterial blood flow in the setting of circulatory collapse. Erroneous DWI and TOF MRA sequence acquisition parameters were ruled out.

Stagnant or absent blood flow augments deoxygenation of blood, that is, a higher oxygen extraction fraction and buildup of intravascular deoxyhemoglobin. As a paramagnetic substance, deoxyhemoglobin acts as an endogenous susceptibility agent, which explains the globally increased conspicuity of cerebral veins on GRE and SWAN images [3]. This characteristic has been employed in stroke imaging as a surrogate of reduced territorial blood flow [3]. On the other hand, the low signal donated by the major cerebral arteries was likely the result of ongoing thrombosis and thrombus-related susceptibility. Other conventional MR sequences (e.g., T2WI and FLAIR) are less sensitive to acute ischemia, given the longer time required for ischemia-related anatomic changes to appear [4].

Our patient’s imaging findings can be simulated by superficial siderosis. Although blood-degradation products are limited to brain surfaces and the meninges, extensive resultant susceptibility on T2*-weighted sequences may produce similar appearances [5]. However, intravascular susceptibility is not a feature of superficial siderosis.

Global increased cerebral vascular susceptibility has been reported in patients scanned following intravenous iron therapy [6]. In our case, the patient did not receive iron, and the lack of flow on MRA helped differentiate. In conclusion, we report a case of hyperacute brain MRI findings in the setting of developing acute circulatory collapse.

## Conclusions

SCA is the abrupt loss of cardiac function that results in acute cardiovascular collapse and subsequent decreased or loss of various organ perfusion. This is the first case describing the MRI imaging findings of ongoing PEA cardiac arrest while being scanned on MRI. Our case is unique in imaging findings, which are not routinely encountered in day-to-day practice. This case raised awareness among the readers.
